# Fatty Degenerative Osteonecrosis of the Jaw: Bridging Molecular Insights and Clinical Practice—A Scoping Review

**DOI:** 10.3390/ijms26051853

**Published:** 2025-02-21

**Authors:** Marzena Dominiak, Wojciech Niemczyk, Artur Pitułaj, Witold Świenc, Jacek Matys

**Affiliations:** 1 Department of Dental Surgery, Faculty of Dentistry, Wroclaw Medical University, Krakowska 26, 50-425 Wroclaw, Poland; marzena.dominiak@umw.edu.pl (M.D.); artur.pitulaj@umw.edu.pl (A.P.);; 2 Medical Center of Innovation, Wroclaw Medical University, Krakowska 26, 50-425 Wroclaw, Poland

**Keywords:** adipocytes, bone marrow, cytokines, inflammation, jaw, osteolysis, osteonecrosis

## Abstract

Fatty degenerative osteonecrosis of the jaw (FDOJ) is a chronic, aseptic inflammatory condition that is characterized by molecular disruptions in bone metabolism and necrotic bone marrow within the jawbone cavities. In contrast to the overt clinical signs typically observed in osteopathies, FDOJ frequently presents with a “silent inflammation” phenotype. The electronic databases PubMed, Scopus, and Embase were searched using appropriate search terms, and the methodology was performed according to PRISMA-ScR guidelines. The elevated expression of inflammatory mediators, particularly C-C motif Chemokine Ligand-5/Regulated on Activation, Normal T Cell Expressed and Secreted (CCL5/RANTES), fibroblast growth factor-2, and interleukin-1 receptor antagonist, distinguishes FDOJ at the molecular level and links it to systemic inflammatory and autoimmune diseases. These immunohistochemical markers play a pivotal role in the pathogenesis of chronic inflammation, immune response regulation, and abnormal bone remodeling. Advanced diagnostic tools, such as conebeam computed tomography and trans-alveolar ultrasonography, facilitate the detection of pathological changes that are not easily discernible with conventional radiography. Surgical intervention remains the primary treatment modality, often complemented by therapies that target these molecular pathways to modulate chronic inflammation. This article underscores the importance of integrating molecular diagnostics, advanced imaging, and clinical data for effective FDOJ detection and management.

## 1. Introduction

Fatty degenerative osteonecrosis of the jaw (FDOJ) is an inflammatory defect characterized by the formation of jawbone cavitations containing necrotic bone marrow. This process can manifest as a silent and asymptomatic condition, which has been described by many as silent or subclinical inflammation [1]. One specific feature of FDOJ allows it to be distinguished from other osteopathies, such as acute and chronic osteomyelitis. Unlike these conditions, FDOJ is painless and is often diagnosed spontaneously during routine screenings in dental offices [1,2]. The first cases of avascular necrosis of the medullary bone were reported at the beginning of the 20th century by the scientist G.V. Black (1915). The initial description of the pathology associated with adipose tissue replacement of healthy bone marrow was presented by Bouquot (1990) as neuralgia-inducing cavitational osteonecrosis (NICO), while examining specimens, obtained from numerous oral surgeons, which contained fatty osteonecrosis from patients experiencing facial neuralgia. A review of the literature reveals the existence of numerous other terms, including FDO, BMDJ, and FDOJ [3].

Despite the fact that the disease is referred to by a number of different names in the medical literature, it is not explicitly listed under a unique term in the International Classification of Diseases (ICD-10). However, according to Lechner, this condition can be classified under the code M87.0—Idiopathic aseptic necrosis of bone [4].

Clinicians may also refer to it as “avascular bone necrosis” or “ischemic aseptic bone necrosis”. Nevertheless, numerous researchers have identified and documented cases of FDOJ, which present with specific symptoms at both the macro- and microscopic levels [1,2,3,4,5,6,7,8,9,10,11,12,13,14,15,16,17,18,19,20,21,22,23,24,25,26,27,28,29,30,31,32,33,34,35,36]. At the macroscopic level, bone samples obtained from affected areas have demonstrated structural deterioration, manifesting as a spongy texture that facilitates the extraction of bone marrow. This phenomenon has been observed in both the maxilla and mandible, presenting as hollow cavitations with soft tissue that has undergone fatty dystrophic alterations. In contrast to the minimal inflammatory cell infiltration observed at this level, there is a notable accumulation of fat cells [14,24,25].

However, the alterations are not limited to the histopathological picture. Notably, significant alterations can also be observed in immunohistochemical tests. Increased inflammatory mediator levels have been identified in FDOJ, with RANTES/CCL5 (chemokine ligand 5) representing a notable example [37]. RANTES/CCL5, a pro-inflammatory cytokine, is produced in elevated amounts by a range of cell types, including platelets, macrophages, eosinophils, and fibroblasts. Additionally, it has been observed in endothelial, epithelial, and endometrial cells in response to various infectious and inflammatory cues. Its primary function is to direct the recruitment of immune cells into damaged or infected tissues [38,39,40].

In light of the findings presented in the existing literature, the authors have formulated a conclusion which analyses and summarizes the current state of knowledge on FDOJ and its link to systemic diseases. Moreover, the range of non-invasive methods is limited, with ultrasound bone measurement emerging as a particularly promising avenue of investigation. The potential benefits and applications of radiological techniques in clinical practice are of significant value in the diagnosis of FDOJ. This paper presents a summary of the current knowledge on this topic, offering guidelines for diagnosis to readers [10,11].

This article aims to address a significant gap in the existing literature by providing a comprehensive review of FDOJ, encompassing its pathophysiology, diagnostic challenges and potential systemic implications. Despite the growing recognition of FDOJ in clinical practice, there remains a dearth of comprehensive reviews that synthesize current knowledge from both a radiological and immunohistochemical perspective. The aim of this study is to emphasize the importance of this condition, investigate the challenges of its diagnosis and explore any potential links to systemic diseases. By synthesizing the available evidence, our aim is to provide clinicians and researchers with a valuable resource to enhance their understanding and management of FDOJ, thereby improving patient outcomes.

## 2. Material and Methods

This article was conducted in accordance with the Preferred Reporting Items for Systematic Reviews and Meta-Analyses (PRISMA) guidelines with an extension for Scoping Reviews (PRISMA-ScR) [41].

### 2.1. Search Strategy

The relevant literature spanning the period from 1982 to 2024 was reviewed using a comprehensive and iterative search strategy, including citation tracking and reference screening. Studies were specifically selected if they incorporated molecular aspects, focusing on mechanisms such as epithelial–mesenchymal transition (EMT), reactive oxygen species (ROS) pathways, and their role in FDOJ. This approach ensured a detailed exploration of immunohistochemical mediators and vitamin D dysregulation, which are pivotal in the pathogenesis of fatty degenerative osteonecrosis of the jaw (FDOJ).

The electronic databases PubMed, Scopus, and Embase were searched using the relevant syntaxes shown in Table 1. In addition, English-language and original paper restrictions were applied.

### 2.2. Study Selection

**Figure 1:**
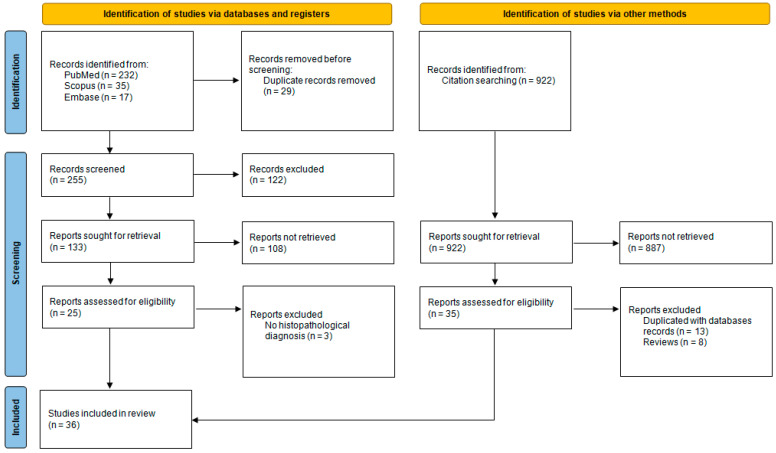
PRISMA 2020 flow diagram.

Following the implementation of the search strategy, a total of 284 articles corresponding to the search phrases used were identified in the three databases. This number was subsequently reduced to 255 after the removal of duplicates. The next stage of the process involved the retrieval of 133 records, from which 25 were assessed for eligibility. A citation search was then performed on the 25 articles using the forward/backward method and the snowball method. A total of 36 most significant articles related to FDOJ were identified and included in the narrative analysis. The flow of the search is shown in Figure 1.

### 2.3. Data Extraction

Among the 2027 patients diagnosed with fatty degenerative osteonecrosis of the jaw, a number of demographic and anatomical patterns were identified. The mean age among the 1929 patients with reported ages was 53.06 years, indicating that FDOJ commonly affects middle-aged individuals. The gender distribution indicated a higher prevalence in women. Of the 1995 patients with recorded gender, 1284 (64.36%) were female, which may suggest a gender predisposition in FDOJ cases.

Anatomical localization data for 280 patients revealed that FDOJ was more frequently observed in the mandible than in the maxilla. In particular, 201 patients (71.79%) exhibited FDOJ lesions in the mandible, while only 79 patients (28.21%) displayed lesions in the maxilla. The lesions were predominantly situated around the third molars or distal regions of the jaw, areas that are particularly prone to trauma and inflammation. This may be due to factors such as wisdom tooth extraction or incomplete molar eruption. This clinical profile highlights both a demographic trend and a common localization pattern, which can facilitate the identification and diagnosis of FDOJ in dental practice(see Table 2).

## 3. Possible Connection to Epithelial Rests of Malassez

Epithelial rests of Malassez (ERMs) have been observed to possess properties analogous to those of stem cells, enabling them to undergo epithelial–mesenchymal transition (EMT) under certain conditions [42]. EMT enables ERMs to differentiate into various cell types, including adipocytes, osteoblasts, and chondrocytes, which may be driven by chronic inflammatory or pathological conditions [42,43]. Such chronic inflammation may have a dentally induced origin [44]. In a study by Schneider et al., the authors showed that in up to 80% of those with FDOJ, the lesion arose at the site of tooth extraction [5]. This differentiation potential indicates that in cases of persistent inflammation, such as in fatty degenerative osteonecrosis of the jaw, ERMs may contribute to the formation of abnormal fatty tissue in the jawbone, leading to the characteristic fatty degeneration observed in these lesions. It is established that ERM cells secrete a number of pro-inflammatory cytokines, including the chemokine RANTES (CCL5), which plays a crucial role in attracting immune cells to sites of inflammation. In FDOJ and analogous conditions, elevated levels of RANTES contribute to the formation of a localized inflammatory environment, which may perpetuate chronic inflammation and disrupt normal bone remodeling processes. The expression of RANTES by ERMs in response to persistent inflammatory stimuli may thus facilitate a cycle of inflammation, tissue degeneration, and abnormal healing, thereby linking ERM activity directly to the pathogenesis observed in FDOJ [45,46]. A detailed description of immunohistochemical parameters will be addressed later in the review.

## 4. The Link Between FDOJ and Reactive Oxygen Species

**Figure 2:**
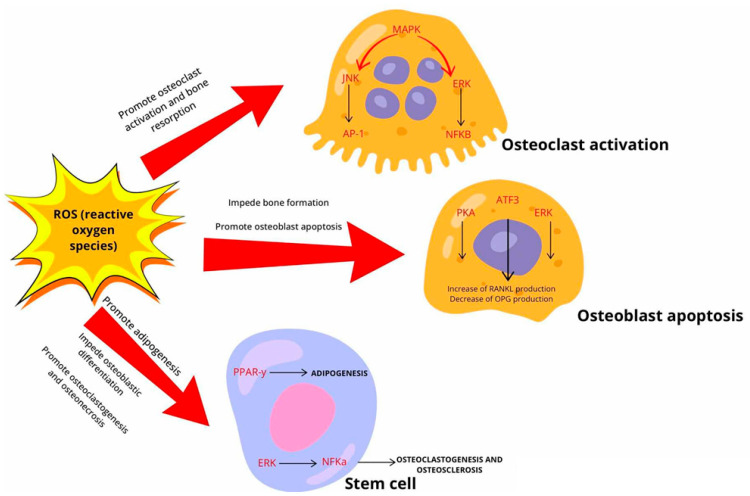
Principal signaling pathways that are disrupted by ROS in osteoblasts, osteoclasts, and MSCs.

Osteoclasts are cells derived from bone marrow macrophages, and play a pivotal role in regulating bone resorption during bone homeostasis. A comprehensive understanding of the factors that regulate osteoclast differentiation and activity is crucial for the study of diseases characterized by an imbalance in bone remodeling, with increased resorption relative to formation. Over the past decade, research has demonstrated that reactive oxygen species (ROS), including superoxide and hydrogen peroxide, play a pivotal role in regulating the differentiation of osteoclasts [47]. There is evidence to suggest that these may play a significant role in the pathogenesis of fatty degenerative osteonecrosis of the jaw. As naturally occurring by-products of cellular metabolism, ROS are involved in normal cellular signaling and processes such as adipocyte differentiation. During the process of adipogenesis, ROS are produced as a consequence of differentiation and as a result of other metabolic processes [48,49]. Nevertheless, the precise sources and mechanisms responsible for ROS production during adipogenic differentiation in mesenchymal stromal/stem cells (MSCs) in vivo remain elusive. Furthermore, it is unclear whether the ROS observed during adipocyte differentiation are a cause or consequence of the process [50]. Notably, antioxidants or ROS scavengers have been shown to suppress ROS effects on adipogenesis, indicating that oxidative stress might influence this differentiation pathway [49,51]. It is established that the primary function of the peroxisome proliferator-activated receptor (PPAR-γ) is to regulate adipocyte differentiation [52]. ROS have been demonstrated to enhance the formation of PPAR-γ via the redox-sensitive nuclear factor (NRF2) pathway [53]. Moreover, evidence indicates that PPAR-γ is capable of inducing an increase in ROS production as well [54,55]. In FDOJ, this oxidative stress has the potential to disrupt bone homeostasis by damaging essential cellular components, including lipids, proteins, and DNA, which could impair bone repair and regeneration. Additionally, elevated ROS levels may stimulate the release of inflammatory cytokines, such as IL-1 and IL-6, contributing to prolonged inflammation and osteonecrosis [49]. The findings of a study by Kanda et al. [56] suggest that an increase in the intracellular ROS level via NAD(P)H oxidase (Nox4) mediates adipocyte differentiation through cAMP response element-binding protein (CREB) in MSCs. Dong et al. observed that free fatty acids (FFAs) released by adipocytes inhibit osteoblast proliferation and function and induce osteoblast apoptosis. Additionally, the same study demonstrated that FFA-generated ROS are a key factor in the adipocyte-induced activation of extracellular signal-regulated kinase (ERK)/P38 signaling, which leads to activation of osteoblast apoptosis [57]. The MAPK family, comprising ERKs, JNKs, and p38, is understood to be activated by MARK kinases, and plays a role in the translocation of c-Fos and NFATc1, which in turn drives osteoclast lineage. It has been demonstrated that ROS inhibit MARK phosphatases, which consequently enhances MARK expression [58]. Additionally, ROS have been observed to activate the β-catenin/FoxOs and ERK/NFκB signaling pathways, while simultaneously inhibiting the p38 signaling pathway, which ultimately results in a reduction in osteoblastogenesis [59]. Figure 2 illustrates the principal signaling pathways that are disrupted by ROS in osteoblasts, osteoclasts, and MSCs.

This indicates the existence of a complex interplay between ROS, osteoclast activation, adipocyte differentiation, and chronic inflammation, which may be the underlying mechanism of FDOJ pathology. Further research is required to elucidate these mechanisms and ascertain whether antioxidant therapy could offer therapeutic benefits for FDOJ by targeting oxidative stress.

## 5. Histopathological Changes

Histopathological examination of FDOJ consistently reveals chronic degenerative changes within the marrow tissue, characterized by fatty degeneration and osteolysis. These changes are the result of ischemic or trophic metabolic insufficiency. A notable finding across studies is the considerable increase in fat cells, frequently accompanied by necrobiotic and mucoid degeneration. Interstitial oedema, myxoid transformation, and the presence of fatty microvesicles and oil cysts are commonly observed, with a notable absence of inflammatory cell responses. The absence of typical inflammatory markers indicates that FDOJ is a form of aseptic inflammation that resembles aseptic ischemic osteonecrosis. Medullary lesions demonstrate widened intertrabecular spaces filled with necrotic bone fragments, liquefied fat, and residual degenerated marrow, which are characteristic of aseptic ischemic osteonecrosis. Alcian blue staining frequently reveals acid mucopolysaccharides, indicative of mucinous changes in the tissue. Additionally, the presence of small nerves in close proximity to necrotic fatty tissue suggests a neuropathic component to the disease [4,21,26,29,34]. Additionally, there is evidence of localized areas of loose fibrosis and mucinogenesis, along with increased bone remodeling of trabeculae. This remodeling indicates an ongoing attempt at tissue repair in response to chronic metabolic disturbance [34]. The collective findings illustrate that FDOJ represents an acute inflammatory response, comparable to that observed in a surgical dental wound, to a chronically inflamed and metabolically disturbed jawbone environment [29]. The aforementioned histopathological features facilitate a more profound comprehension of the progression of FDOJ, and serve to reinforce its distinctive categorization as a non-infectious, aseptic form of inflammation within the jawbone.

## 6. Changes in Immunohistochemical Parameters

### 6.1. C-C Motif Chemokine Ligand-5/Regulated on Activation, Normal T Cell Expressed and Secreted (CCL5/RANTES)

**Figure 3:**
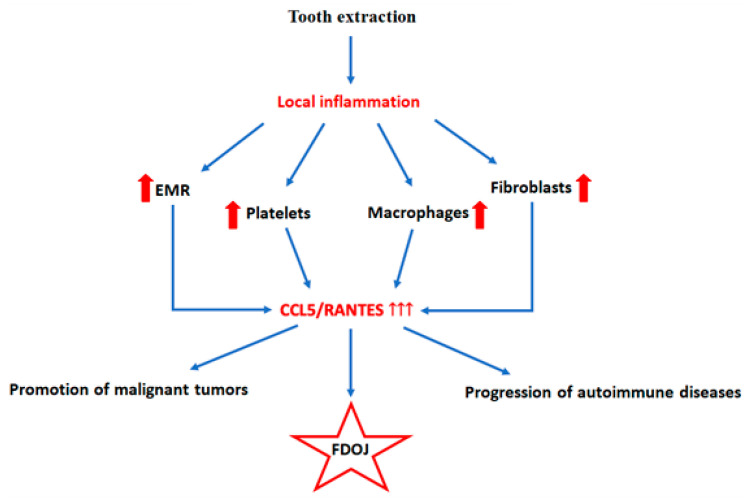
A diagram illustrating the causal sequence of FDOJ formation subsequent to tooth extractions.

CCL5/RANTES is a 68-amino-acid protein belonging to the CC chemokine family, which functions as a signaling molecule in the immune system. CCL5/RANTES plays a significant role in inflammatory processes and immune responses, particularly in attracting specific types of immune cells to sites of inflammation [60]. CCL5/RANTES is released in significant quantities by a number of cell types in response to infectious or inflammatory stimuli, including platelets, macrophages, fibroblasts, and eosinophils, as well as epithelial cells, vascular endothelial cells, and endometrial cells [39]. This recruitment of immune cells to damaged or infected tissues is a key aspect of the body’s immune response. The primary physiological functions of CCL5/RANTES encompass the chemotaxis of T lymphocytes, eosinophils, and basophils to sites of inflammation. Furthermore, it plays a role in the recruitment of natural killer (NK) cells and dendritic cells. Furthermore, it facilitates the maintenance of inflammatory states by stimulating the secretion of inflammatory mediators, such as cytokines [61]. Elevated levels of CCL5/RANTES have been demonstrated to contribute to the pathogenesis and progression of autoimmune disorders, including bronchial asthma, rheumatoid arthritis, systemic lupus erythematosus, and Addison’s disease [62,63,64].Although the inflammatory response of the body represents one of the body’s defense mechanisms against cancerous growths, a chronically sustained inflammatory state can paradoxically promote their development. Elevated levels of CCL5/RANTES have been observed in specific malignant neoplasms, including breast cancer, malignant melanoma, and prostate cancer [65,66,67]. In the context of FDOJ, CCL5/RANTES is a principal inflammatory mediator associated with this condition (Figure 3). A substantial body of clinical evidence demonstrates that RANTES levels in regions affected by FDOJ are markedly elevated, frequently exceeding typical values by several-fold [1,2,4,8,10,11,20,21,22,23,24,25,26,27,28,29,32,33,34].The physiological inflammatory response as a consequence of localized bone tissue injury, such as that resulting from periapical tissue inflammation, bone damage caused by a dental implant, or the state following tooth extraction, can transition into a chronic process. This is accompanied by elevated local levels of RANTES. This prolonged inflammatory process leads to the development of disorders characterized by fatty degeneration of the bone marrow [22]. It is noteworthy that FDOJ frequently coexists with various systemic diseases, including those affecting the nervous system (such as migraines, trigeminal neuralgia, and neurodegenerative diseases), rheumatoid disorders, and cancers (such as breast and prostate cancer). These conditions share a common denominator: elevated levels of CCL5/RANTES [21,23,25,26,28,29].

### 6.2. Fibroblast Growth Factor-2 (FGF-2)

FGF-2 is a key mediator of biological processes such as angiogenesis, fibroblast stimulation, and the regulation of osteoblast proliferation and differentiation [68]. In bone tissue, it plays a significant role in maintaining homeostasis and remodeling [69]. FGF-2 is particularly important in the response to tissue damage, initiating repair processes and supporting the growth of blood vessels in areas of injury [70]. The reviewed literature shows a clear correlation between the diagnosis of FDOJ and elevated levels of FGF-2 in the tissues affected by this pathological process [2,4,8,10,24,25,26,29]. The elevated levels of FGF-2 in FDOJ may result from the body’s compensatory response to chronic inflammation and bone tissue damage. Bone matrix degradation, the secretion of FGF-2 by macrophages and fibroblasts in the inflamed area, and the response to hypoxia associated with the inflammatory process contribute to a significant increase in its levels [71]. For this reason, elevated levels of FGF-2 may serve as a diagnostic marker for identifying FDOJ areas, particularly when accompanied by increased levels of CCL5/RANTES. As noted by Lechner, heightened expression of CCL5/RANTES and FGF-2 correlates with the occurrence of neurodegenerative diseases, rheumatoid arthritis, and breast cancer [24]. Although insufficient data exist to draw definitive conclusions, this observation may suggest a synergistic effect of CCL5/RANTES and FGF-2, stimulated by intrabony changes associated with bone marrow fat degeneration, on adverse interactions with other tissues in the body.

### 6.3. Interleukin-1 Receptor Antagonist (IL-1ra)

This protein acts as a natural inhibitor of interleukin-1 (IL-1), a key mediator of inflammatory responses. IL-1ra binds to IL-1 receptors, blocking their activation and thereby serving a protective role by preventing excessive inflammation that could lead to tissue damage [72]. In the bone environment, as a response to chronic inflammation, IL-1ra primarily blocks IL-1β, leading to a reduction in osteoclast stimulation and the associated excessive bone destruction [73]. Clinical data show that the level of IL-1ra in tissues affected by FDOJ is approximately 3 to 3.5 times higher than in healthy bone. For this reason, IL-1ra may serve as a diagnostic marker, alongside CCL5/RANTES and FGF-2, to aid in identifying patients with inflammatory conditions within the jawbone [2,4,8,10,27].

### 6.4. Interleukin-6 (IL-6) and Tumor Necrosis Factor Alpha (TNF-α)

Both TNF-α and IL-6 are key cytokines that play essential roles in inflammatory, immune, and regenerative processes. Despite their differing functions and mechanisms of action, their combined activity modulates the immune response. TNF-α is a potent inflammatory mediator produced by macrophages and monocytes. Its primary action is to induce an acute inflammatory response by binding to TNFR-1 and TNFR-2 receptors, activating NF-κB pathways, and leading to apoptosis [74]. IL-6 is a pleiotropic cytokine. In the acute inflammatory response, IL-6 activates TNF-α and neutrophils, stimulates macrophage maturation, and promotes the differentiation of Tc lymphocytes [75,76,77]. By recruiting monocytes to areas affected by acute inflammation, IL-6 contributes to the transition from an acute to a chronic state. This makes its physiological function the prolongation of the inflammatory response and support of systemic reactions [78]. In tissues affected by FDOJ, a reduction in the concentration of the described cytokines has been observed [2,27]. This indicates the low immunogenicity of such pathological changes, which often exhibit a limited ability to trigger a response associated with clinical symptoms such as pain or swelling. Low concentrations of TNF-α and IL-6 may explain the sparse or even absent clinical symptoms, leading to delays in diagnosis and the initiation of appropriate treatment.

### 6.5. Vitamin D

FDOJ has been associated with disturbances in vitamin D metabolism, particularly with regard to the deactivation of the vitamin D receptor (VDR). The VDR plays a pivotal role in regulating both immune responses and bone metabolism, including the expression of antimicrobial peptides and the equilibrium between osteoclast and osteoblast activity [79]. In FDOJ, the deactivation of the VDR impairs the body’s capacity to effectively regulate inflammation, thereby creating a chronic, “silent” inflammatory environment in the jawbone. This dysregulation results in increased production of active 1,25-dihydroxyvitamin D3 (1,25D) and a concurrent decrease in 25-hydroxyvitamin D3 (25D), a pattern that is associated with chronic inflammation. Elevated levels of 1,25D have been observed to promote osteoclast activity and bone resorption, which contribute to the characteristic fatty degeneration and trabecular osteolysis seen in FDOJ lesions. Additionally, the overexpression of RANTES/CCL5 in FDOJ regions intensifies local inflammation and impedes normal wound healing processes. This feedback loop between VDR deactivation, cytokine dysregulation, and bone metabolism underscores the intricate interplay of systemic and local factors driving FDOJ pathogenesis. These findings suggest that FDOJ represents a distinctive form of aseptic osteonecrosis influenced by systemic vitamin D metabolism abnormalities and chronic inflammation [29]. A summary of changes in immunohistochemical parameters in FDOJ is presented in Table 3.

## 7. Imaging Modalities of FDOJ

### 7.1. Orthopantomograms (OPGs) and Periapical Radiographs (PRs)

A radiological examination represents a fundamental tool in the management of patients. Dental diagnostics and treatment planning, in addition to monitoring treatment outcomes and complications, are dependent on both clinical examination and diagnostic imaging. Periapical radiographs and OPGs are both associated with inherent limitations, such as the superimposition of anatomical structures, geometrical distortion, anatomical noise, and a two-dimensional representation [80]. FDOJ is typically observed as poorly defined or indistinct radiolucent zones, frequently accompanied by reduced trabecular opacity within the lesion. The aforementioned features may vary significantly, with some lesions presenting as moderately or sharply demarcated radiolucencies. In a considerable number of cases, FDOJ lesions manifest in regions where prior tooth extractions have occurred, exhibiting distinctive alterations in the processes of bone healing. The radiographic findings frequently overlap with those of other osteolytic conditions, thereby rendering a diagnosis based solely on OPG or periapical films challenging [5]. It has been demonstrated that OPG frequently fails to accurately reflect the true extent of FDOJ. Lesions may be undetectable by radiography in the early stages or may manifest as minor irregularities, resulting in a false-negative diagnosis. This is of particular importance, given that FDOJ frequently presents without overt clinical symptoms, thereby rendering radiographs an unreliable standalone diagnostic tool [1]. A biopsy may be necessary for a definitive diagnosis to be reached. The difficulty in distinguishing FDOJ from other lesions highlights the necessity for supplementary imaging techniques, such as conebeam computed tomography (CBCT) or advanced methods like trans-alveolar ultrasonography (TAU), which offer more comprehensive evaluations of bone density and structure. The lack of distinct radiological markers makes these lesions challenging to differentiate without further analysis [1,5].

### 7.2. Computer Tomography (CT) and Digital Volume Tomography (DVT)

Digital volume tomography (DVT) is a type of cone beam computed tomography that allows for the generation of high-quality three-dimensional images of osseous structures. It is a well-established diagnostic tool in the field of dentistry. The use of DVT enables the attainment of high detail resolution, while simultaneously reducing the radiation dose exposure in comparison to that of conventional computed tomography [81]. DVT offers substantial benefits in the evaluation of FDOJ. This imaging technique generates three-dimensional representations of jawbone structures, facilitating superior resolution and the capacity to quantify bone density through the utilization of Hounsfield Units (HU). Three-dimensional (3D) imaging techniques (Figure 3) such as DVT effectively overcome the inherent limitations of two-dimensional (2D) imaging by eliminating superimpositions and geometrical distortions, thereby facilitating detailed visualization of both trabecular and cortical bone. In FDOJ, DVT reveals areas of diminished density, often below 150 HU, which are indicative of bone marrow degeneration and fatty osteonecrosis. Furthermore, DVT is capable of differentiating healthy bone (above 300 HU) from pathological changes, thereby facilitating more accurate localization and evaluation of FDOJ lesions [1,24,25,26,27,34].

### 7.3. Transmission Alveolar Ultrasonography

Trans-alveolar ultrasonography represents a novel imaging modality that has been devised for the assessment of jawbone density and the detection of focal bone marrow defects, including fatty degenerative osteonecrosis of the jaw. TAU employs the use of ultrasound waves transmitted through the alveolar ridge, offering a non-invasive and radiation-free diagnostic alternative. The method employs a dedicated device, designated as TAU-n, which incorporates an external transmitter and an internal receiver arranged in a coplanar configuration. These components are designed to work in conjunction with one another in order to measure the degree of attenuation of ultrasound waves as they traverse the jawbone. The degree of attenuation is indicative of variations in bone density, thereby facilitating the identification of pathological areas [1]. FDOJ lesions display distinctive alterations, including fatty degeneration and reduced mineralization. These are then visualized on the TAU display as regions of high attenuation, which correspond to diminished bone density. A color-coded scale is employed to represent different densities, with green or white indicating healthy bone, and red or black signifying areas with severely compromised mineralization, which is typical of FDOJ [1,2,8,20,22,24,25,26,27,31,33,34]. TAU measurements can be represented numerically, with higher attenuation values correlating with pathological bone conditions [1].

## 8. Clinical Symptoms

Intrabony defects characterized by fatty degeneration of bone marrow are most commonly described as asymptomatic, with the mucosa covering the affected bone being free of pathological changes, including signs of infection [14,16,17]. Due to the extremely rare clinical symptoms, likely caused by a specific immune response involving elevated IL-1ra levels and decreased TNF-alpha and IL-6 levels, FDOJ is sometimes referred to as “silent inflammation” [2,4,27]. These changes are associated with edentulous areas where tooth extraction has occurred in the past [31]. No clinical signs of bone expansion are observed [17,82]. In 1982, Lipani et al. described cases of “hematopoietic bone marrow defects” presenting clinical symptoms such as swelling, pain, and cortical plate expansion. Although these changes showed microscopic evidence of fat cells, there was no hyperplasia of these cells [12]. Additionally, chemokine levels, an important diagnostic factor, were not assessed. Therefore, despite their association with bone marrow disorders, the cases described by Lipani likely do not directly relate to FDOJ changes. FDOJ, on the other hand, is often associated with systemic diseases, such as inflammatory and neurodegenerative diseases of the central nervous system or autoimmune disorders, which are linked to high levels of CCL5/RANTES in the body. One such condition may be Chronic Fatigue Syndrome, whose typical clinical symptoms can suggest the presence of FDOJ [27]. Degeneration of bone marrow associated with prolonged inflammatory processes may also manifest as NICO, which has the ability to cause neuralgic pain [83]. However, the histopathological appearance of these changes is not identical to FDOJ, and they are not accompanied by the proliferation of fatty tissue [3]. In exceptional cases, fatty bone marrow lesions can be detected intraoperatively, such as during the placement of a dental implant. Bone affected by fatty degeneration exhibits reduced density and weakened mechanical properties, directly leading to difficulties in achieving proper primary stability. Lee et al. describe extreme cases of distant displacement of implants in the region of lower first molars within the mandibular body, requiring surgical removal [6]. The softening of the FDOJ medulla is so pronounced that the bone marrow space can indeed be suctioned or spooned out [4].

## 9. Treatment

As the primary diagnostic tool for intramedullary changes involves nonspecific radiographic examinations, FDOJ should initially be differentiated from other similar lesions, including locally aggressive conditions such as residual cysts, traumatic bone cysts, aneurysmal bone cysts, and central giant cell granulomas, as well as other tumors and primary or metastatic malignancies [16]. Compared to most of these conditions, FDOJ statistically occurs more frequently in middle-aged women suffering from the aforementioned chronic diseases, primarily affecting the nervous and immune systems or cancer. In such individuals, laboratory tests such as serum Th1/Th2 profile evaluation are advisable [27]. Unfortunately, even data obtained from patient profiling and additional tests do not guarantee certainty about the true nature of the lesion. Diagnostic difficulties necessitate definitive diagnosis based on histopathological examination and the identification of characteristic microscopic features in the affected area [16]. Therefore, surgical treatment remains the first-line approach. Surgical removal of FDOJ lesions contributes to reducing the source of CCL5/RANTES synthesis, but may not be sufficient to re-establish altered immunological patterns, especially in patients suffering from other CCL5/RANTES-dependent diseases. Hence, supplementing surgical therapy with an immunomodulating RANTES 27CH medication shows promising results [28]. Due to the absence of bacteria in FDOJ lesions, antibiotic therapy is ineffective [33].

## 10. Discussion and Conclusions

Fatty degenerative osteonecrosis of the jaw represents a complex and under-recognized condition, characterized by chronic, aseptic inflammation and pathological bone marrow degeneration. The condition’s silent nature and subtle clinical presentation present significant diagnostic challenges. The absence of overt symptoms, such as pain or visible swelling, highlights the necessity for advanced imaging and immunohistochemical analyses in the detection of FDOJ. Despite their widespread use, traditional diagnostic tools such as orthopantomograms and periapical radiographs often prove inadequate for identifying early-stage or subtle lesions. This necessitates the use of supplementary methods, including CBCT and trans-alveolar ultrasonography.

Histopathological findings consistently reveal fatty degeneration, osteolysis, and an increased presence of adipocytes in the affected bone marrow, without the typical inflammatory cell responses observed in other conditions. This distinctive pathology serves to highlight the aseptic nature of FDOJ, thereby differentiating it from other conditions affecting the jawbone, such as osteomyelitis. The involvement of pro-inflammatory mediators, particularly elevated levels of RANTES/CCL5, FGF-2, and IL-1ra, indicates a crucial association between FDOJ and systemic inflammatory conditions, including autoimmune diseases and specific types of cancer. These immunohistochemical markers not only improve diagnostic precision, but also present potential targets for future therapeutic interventions.

This study has been conducted with rigor and follows PRISMA-ScR guidelines; however, there are several limitations that must be considered when interpreting the results. Firstly, the broad literature search period (1982–2024) encompasses evolving diagnostic criteria, imaging technologies, and treatment methodologies, leading to inconsistencies in findings. Secondly, the selection of studies was narrative rather than systematic, which may introduce selection bias and limit reproducibility. The heterogeneity of the included studies, in terms of methodologies, patient populations, and diagnostic approaches, complicates the establishment of direct comparisons and affects the generalizability of the conclusions. Another limitation is the absence of standardized diagnostic criteria for FDOJ, with different studies using varying terminology and classification systems, which complicates the interpretation of results and underscores the need for universally accepted diagnostic guidelines. Furthermore, while this study highlights the immunohistochemical and radiological aspects of FDOJ, long-term clinical and treatment outcome data remain scarce. The potential links between FDOJ and systemic diseases, such as autoimmune and neurodegenerative conditions, require further high-quality, prospective studies to establish causative relationships, rather than mere associations.

Furthermore, while the utilization of advanced imaging techniques such as CBCT and trans-alveolar ultrasonography is commendable, their accessibility in all clinical settings is limited, thus hindering their applicability for routine diagnosis. Surgical intervention remains the primary treatment modality; however, there is a paucity of consensus on standardized treatment protocols, including the role of adjunctive therapies. Furthermore, the majority of studies reviewed were observational or retrospective, emphasizing the necessity for controlled clinical trials to validate findings and develop evidence-based diagnostic and therapeutic strategies for FDOJ.

In the future, the incorporation of sophisticated imaging methodologies and molecular diagnostics will be pivotal in enhancing the early detection and management of FDOJ. Digital volume tomography and TAU have the potential to provide detailed, three-dimensional assessments of bone integrity and density. Furthermore, additional research is required to investigate the systemic implications of FDOJ, particularly its relationship with chronic inflammatory and neurodegenerative diseases. The development of targeted therapies that address the underlying inflammatory processes, such as immunomodulatory treatments, may offer new avenues for effective management.

In conclusion, a multidisciplinary approach integrating radiological, immunohistochemical, and clinical data is essential for a comprehensive understanding of FDOJ. Continued research and collaboration across dental and medical fields will be pivotal in elucidating new insights into the pathogenesis, diagnosis, and treatment of this hitherto under-researched yet impactful condition.

## Figures and Tables

**Table 1:** Syntaxes for individual databases.

Database	Search Terms	Results
PubMed	(“Fatty Degenerative Osteonecrosis of the Jaw” OR “FDOJ” OR “Aseptic Osteomyelitis” OR “Avascular Osteonecrosis” OR “Ischemic Osteonecrosis” OR “Ischemic Bone Disease” OR “Chronic Ischemic Jawbone Disease” OR “CIBD” OR “Hole in the Jaw Bone” OR “Bone Marrow Oedema” OR “Regional Ischemic Osteoporosis” OR “Osteomyelitis” OR “Osteonecrosis” OR “Chronic Osteitis” OR Ratner’s Cyst” OR “Robert’s Cyst” OR “Osteocavitations” OR “Bone Cavitations” OR “Neuralgia-inducing Cavitational Osteonecrosis” OR “NICO”) AND (‘‘ Oral” OR “Mouth” OR “Dentistry” OR “Dental”).	232
Scopus	TITLE-ABS-KEY(“fatty degenerative osteonecrosis”) OR TITLE-ABS-KEY(“fatty degenerative osteolysis”) OR (TITLE-ABS-KEY(“bone marrow defect”) AND TITLE-ABS-KEY(“jawbone”)) OR (TITLE-ABS-KEY(“bone marrow defect”) AND TITLE-ABS-KEY(“maxilla”)) OR (TITLE-ABS-KEY(“bone marrow defect”) AND TITLE-ABS-KEY(“jaw”)) OR (TITLE-ABS-KEY(“bone marrow defect”) AND TITLE-ABS-KEY(“mandible”))	35
Embase	(’fatty degenerative osteonecrosis’ OR ’fatty degenerative osteolysis’) OR (’bone marrow defect’ AND (’jawbone’ OR ’maxilla’ OR ’jaw’ OR ’mandible’)) OR (’bone marrow defect’:ti,ab AND (’jaw’:ti,ab OR ’mandible’:ti,ab))	17

Limitations: original paper, English language.

**Table 2:** Generalized characteristics of included studies.

Authors/Year	Terminology Used	Participants	Average Age of Patients	Region of Bone Lesion	Immunohistochemical Diagnostics	Radiological Diagnostics
**Lechner et al. (2021) [1]**	BMDJ/FDOJ	210 (F:M 129/81)	53.02	Jawbones	R/C = Rantes	OPG, DVT, USG (Tau)
**Lechner et al. (2018) [29]**	FDOJ	43 (F:M 25:18)	54.05	Jawbones	FGF-2, IL-1ra, R/C = RANTES	No data
**Floris et al. (2018) [28]**	FDOJ	46 (F:M 28:18)	No info	Jawbones	R/C = RANTES	No data
**Lechner et al. (2024) [27]**	BMDJ/FDOJ	128 (F:M 86:42)	54	Jawbones	IL-1ra, IL-6, TNF-alfa, R/C = RANTES	OPG, DVT, USG (Tau)
**Lechner et al. (2021) [26]**	BMDJ/FDOJ	17 (F:M 13:4)	53	Jawbones	R/C = RANTES, FGF-2, IL-1ra	OPG, DVT, USG (Tau)
**Lechner and Von Baehr (2014) [25]**	FDOJ	23 (F:M 23:0)	60.5	Jawbones	R/C = RANTES, FGF-2, IL-1ra	OPG, DVT, USG (Tau)
**Lechner et al. (2013) [24]**	JC/NICO	31 (F:M 20:11)	57.1	15 maxilla, 16 mandible	R/C = RANTES, FGF-2, IL-1ra	OPG, DVT, USG (Tau)
**Lechner et al. (2021) [23]**	BMDJ	301 (F:M 89:225)	54.05	Jawbones	R/C = RANTES	OPG, DVT
**Diederich et al. (2023) [22]**	BMDJ	113 (F:M 70:43)	47.58	Jawbones	R/C = RANTES	CT, DVT, USG (Tau)
**Lechner et al. (2019) [21]**	FDOJ	449 (F:M 298:151)	53.02	Jawbones	R/C = RANTES	OPG, CT, DVT
**Lechner and Schick (2021) [20]**	BMDJ	1 (sex not revealed)	28	Posterior mandible	R/C = RANTES	OPG, DVT, CBCT, USG (Tau)
**Gordy et al. (1993) [19]**	FOBMD	1 F	56	Anterior right maxilla	No data	Periapical radiograph, panoramic
**Goldlatt et al. (2017) [18]**	CFOJ	227 (F:M 198:29)	53	Jawbones	No data	Periapical radiograph, CBCT, Tech 99 bone scan
**Almeida et al. (2014) [17]**	FOBMD	1 F	66	Posterior mandible	No data	OPG, CT
**Bravo-Calderon et al. (2012) [16]**	OBMD	1 F	32	Body of mandible	No data	OPG
**Sençimen et al. (2011) [15]**	FOBMD	1 F	35	Right side of mandible	No data	Periapical radiograph, OPG
**Sa’Do et al. (1992) [14]**	OBMD	1 F	28	Mandible	Serum chemistry, CBC, ESR, CRP, Iron, Transferrin	OPG, CT
**Basugi et al. (2020) [13]**	FOBMD	1 M	>70	Mandible	No data	OPG, CT, MR
**Lipani et al. (1982) [12]**	HDJ	16 (F:M 15:1)	42.75	Posterior mandible (15) and maxillary tuberosity (1)	No data	Two-dimensional radiograph
**Bouquot et al. (1992) [3]**	NICO	2:1 (F:M)	49.1	Mandibular and maxillary molar areas and maxillary cuspid region	No data	Authors worked on tissue samples
**Lechner et al. (2021) [11]**	FDOJ	39 F	49.8	Jawbones	RANTES	3D-DVT, 2D-OPG
**Lechner and Von Baehr (2015) [10]**	FDOJ	15 (14:1) F:M	60	Mandibular alveolar bone	RANTES, FGF 2, IL-1ra, MCP-1	OPG, peripheral radiograph
**Medeiros et al. (2016) [9]**	FOBMD	1 F	52	Right maxillary area	No data	CBCT
**Lechner et al. (2018) [8]**	FDOJ	14 (F:M 13:81)	54	Jawbones	RANTES, FGF-2, IL-1ra	OPG, CBCT, TAU
**Barker et al. (1974) [7]**	FOHBMD	1 F	35	Right side of mandible	No data	Peripheral radiograph, OPG
**Makek and Lello (1986) [30]**	FOBMD	20 (7:13 M:F)	Between 30 and 50	Mandibular molar area	No data	Peripheral radiograph
**Shankland and Bouquot (2004) [31]**	FOMD	100 (77:23 F:M)	50.15	35% in maxilla, 65% in mandible	No data	TAU, OPG
**Chiang et al. (2015) [32]**	FOMD	1 F	28	Maxilla	No data	CBCT, OPG
**Lechner et al. (2017) [4]**	AIOJ/FDOJ	24	No data	9 maxilla, 15 mandible	FGF-2, IL-1ra, IL-6, IL-8, MCP-1, TNF-alfa, R/C	OPG
**Vasconcelos E Cruz et al. (2024) [33]**	BMDJ/FDOJ	45 (7 with FDOJ)	No data	Jawbones—not specified	R/C = RANTES	OPG, CBCT/DVT, TAU
**Lechner et al. (2020) [34]**	FOBMD	82 (F:M 59:23)	56.4	Jawbones—not specified	RANTES/CCL5	DVT/CBCT, TAU
**Lechner et al. (2017) [2]**	FDOJ	40 (21 with FDOJ) (F:M 8:13)	56.4	11 maxilla, 10 mandible	RANTES/CCL5, FGF-2, IL-1ra, IL-6, IL-8, MCP-1, TNF-alfa	OPG, DVT, TAU
**Ida-Yonemochi et al. (2010) [35]**	FOBMD	1 M	9	1 maxilla	No data	Periapical radiograph
**Wilson et al. (1985) [36]**	FOBMD	3 (F:M 1:2)	49.3	3 mandible	No data	OPG, Periapical radiograph
**Schneider et al. (1988) [5]**	OBMD	20 (F:M 14:4)	43	4 maxilla, 16 mandible	No data	OPG, Periapical radiograph
**Lee et al. (2013) [6]**	FOBMD	3 F	54	3 mandible	No data	CBCT, OPG

BMDJ—bone marrow defect of the jaw, FDOJ—fatty osteonecrosis of the jaw, OPG—orthopantomogram, DVT—digital volume tomography, USG—ultrasonography, F—female, M—male, IL-1ra—interleukin-1 receptor antagonist, IL-6—interleukin-6, TNF-alfa—tumor necrotic factor-alfa, JC—jawbone cavitation, NICO—neuralgia-inducing cavitationalosteonecrosis, FOHBMD—Focal Osteoporotic Hematopoietic Bone Marrow Defect, FGF-2—fibroblast growth factor-2, CT—computer tomography, CBCT—cone beam computer tomography, HDJ—Hemoatopoetic Defect of the Jaw, FOBMD—Focal Osteoporotic Bone Marrow Defect, FOMD—Focal Osteoporotic Marrow Defect, AIOJ—aseptic-avascular osteonecrosis, CFOJ—chronic fibrosing osteomyelitis of the jaw, OBMD—osteoporotic bone marrow defect, MR—magnetic resonance, CCL5—C-C motif Chemokine Ligand-5, RANTES—Regulated on Activation, Normal T Cell Expressed and Secreted, TAU—Transmission Alveolar Ultrasonography.

**Table 3:** Immunohistochemical changes in FDOJ.

Parameter	Function	Change in FDOJ	Key Notes
**CCL5/RANTES**	- Attracts immune cells to inflammation sites. - Sustains inflammation by cytokine release.	Elevated	- Linked to systemic diseases (e.g., neurodegenerative disorders, cancers). - Diagnostic marker for FDOJ.
**FGF-2**	- Promotes angiogenesis, fibroblast activation, and osteoblast regulation. - Responds to tissue damage and hypoxia.	Elevated	- Reflects chronic inflammation. - Combined with CCL5/RANTES as diagnostic marker. - Associated with systemic diseases.
**IL-1ra**	- Inhibits IL-1, reducing osteoclast activity and tissue damage.	Elevated	- Indicates chronic inflammation. - Diagnostic marker alongside CCL5/RANTES and FGF-2.
**IL-6**	- Transition from acute to chronic inflammation. - Recruits monocytes and supports systemic responses.	Reduced	- Reflects low immunogenicity. - Explains sparse clinical symptoms and delays in diagnosis.
**TNF-α**	- Initiates acute inflammation via NF-κB pathway activation.	Reduced	- Indicates low inflammatory activity in FDOJ regions. - Contributes to lack of pain and swelling in clinical presentation.
**Vitamin D Metabolism**	- Regulates immune and bone homeostasis. - Balances osteoclast and osteoblast activity.	Dysregulated**:↑** 1,25D**↓** 25D	- VDR deactivation creates chronic “silent” inflammation. - Elevated osteoclast activity leads to bone resorption. - Contributes to FDOJ pathogenesis.

FDOJ—fatty degenerative osteonecrosis of the jaw, FGF-2—fibroblast growth factor-2, IL-1ra—interleukin-1 receptor antagonist, IL-6—interleukin-6, TNF-α—tumor necrosis factor alpha, 1,25D—1,25-dihydroxyvitamin D3, 25D—-hydroxyvitamin D3.
